# SPHK/HIF-1α Signaling Pathway Has a Critical Role in Chrysin-Induced Anticancer Activity in Hypoxia-Induced PC-3 Cells

**DOI:** 10.3390/cells11182787

**Published:** 2022-09-07

**Authors:** Hengmin Han, Seon-Ok Lee, Yinzhu Xu, Jung-Eun Kim, Hyo-Jeong Lee

**Affiliations:** 1 Department of Cancer Preventive Material Development, College of Korean Medicine, Graduate School, Kyung Hee University, 26, Kyungheedae-ro, Dongdaemun-gu, Seoul 02447, Korea; 2 Department of Science in Korean Medicine, College of Korean Medicine, Graduate School, Kyung Hee University, 26, Kyungheedae-ro, Dongdaemun-gu, Seoul 02447, Korea

**Keywords:** prostate cancer, sphingosine kinase 1, hypoxia-inducible factor-1α, PC-3 xenograft model, chrysin

## Abstract

Hypoxia, a typical feature of locally advanced solid tumors including prostate cancer, is a critical contributor to tumor progression and causes resistance to therapy. In this study, we investigated the effects of chrysin on tumor progression in hypoxic PC-3 cells. Chrysin exerted a significant inhibitory effect on 3D cell growth under normoxic and hypoxic conditions. It also decreased the hypoxia-induced vasculogenic mimicry and attenuated the expression of HIF-1α and VE-cadherin. Chrysin inhibited HIF-1α accumulation in a concentration- and time-dependent manner in hypoxic PC-3 cells, while also suppressing the expression of HIF-1α by inhibiting SPHK-1 in both CoCl_2_ and hypoxic PC-3 cells. At high concentrations of chrysin, there was a greater increase in apoptosis in the hypoxic cells compared to that in normoxic cells, which was accompanied by sub-G1 phase arrest. Chrysin-induced apoptosis inhibited VEGF and Bcl-2 and induced the cleavage of PARP and caspase-3. SPHK-1 knockdown induced apoptosis and inhibited epithelial–mesenchymal transition. Consistent with the in vitro data, 50 mg/kg of chrysin suppressed the tumor growth of PC-3 xenografts by 80.4% compared to that in the untreated control group. The immunohistochemistry of tumor tissues revealed decreased Ki-67, HIF-1α, and VEGF expression in the chrysin-treated group compared to an untreated control. Western blotting data for tumor tissues showed that chrysin treatment decreased SPHK-1, HIF-1α, and PARP expression while inducing caspase-3 cleavage. Overall, our findings suggest that chrysin exerts anti-tumor activity by inhibiting SPHK-1/HIF-1α signaling and thus represents a potent chemotherapeutic agent for hypoxia, which promotes cancer progression and is related to poor prognoses in prostate cancer patients.

## 1. Introduction

Prostate cancer is the second most common cancer in males [1]. Most patients with prostate cancer (nearly 80%) are diagnosed early and cured by treatments that most often involve radiation or surgery. However, approximately 20% of the patients diagnosed with prostate cancer have a more aggressive form of the disease [2]. Hypoxia is a considerably early event during prostate carcinogenesis and is responsible for the aggressive phenotype of prostate cancer growth, promotion, metastasis, and hormone-refractory progression. Hypoxia is a common characteristic of locally advanced solid tumors [3] and up to 50–60% of solid tumors include areas of hypoxic tissue [4]. In prostate cancer, hypoxia contributes to high Gleason scores and has been implicated in the emergence of castration-resistant prostate cancer cells [5,6]. Vasculogenic mimicry (VM), which is promoted by hypoxia, is a pivotal characteristic of the tumor microenvironment during tumor progression [7]. In a recent study, VM was found to be associated with high Gleason scores in 92 prostate cancer tissues [8]. VM was initially discovered in melanoma and has subsequently been observed in various tumors including hepatocellular carcinoma, gastric adenocarcinoma, gallbladder carcinoma, ovarian cancer, and prostate cancer [9,10,11,12]. VM is the de novo formation of vessel-like networks by aggressive tumor cells induced by hypoxia. It provides sufficient oxygen and nutrients to sustain tumor survival and growth and connects with normal or leaky blood vessels [13,14,15].

The most commonly used cell lines for prostate cancer research are LNCaP, PC-3, and DU145. The LNCaP cell line is androgen-sensitive human prostate cancer cell line, and PC-3 and DU145 cells are androgen-independent prostate cancer cell lines. PC-3 cells among these cell lines have the most metastatic ability and belong to a castrate-resistant prostate cancer-like cell line. It has been reported that the basal hypoxia-inducible factor-1α (HIF-1α) protein level was the highest expressed in PC-3 cells [16].

HIF-1α is a critical transcription factor that regulates a large number of genes related to tumor progression, such as tumor growth, invasion, and metastasis [17,18]. HIF-1α is highly expressed in various solid cancers including prostate cancer. Under normoxic conditions, HIF-1α becomes prolyl hydroxylated and ubiquitylated by the tumor suppressor von Hippel–Lindau protein (pVHL) of the E3 ligase complex, thus becoming degraded in proteasomes. Under hypoxic conditions, the prolyl hydroxylase activity is attenuated and the HIF-1α protein stabilized [19,20,21]. Hypoxia-induced HIF-1α stability also involves the AKT/glycogen synthase kinase 3 (GSK3) pathway, which acts downstream of sphingosine kinase-1 (SPHK-1) [22,23].

The key mechanism identified in this study involved an anticancer mechanism that regulates tumor growth, VM, tumor proliferation, apoptosis, and reactive oxygen species (ROS) by inhibition of the SPHK-1/HIF-1α pathway. SPHK-1 is an oncogenic lipid kinase that catalyzes the phosphorylation of sphingosine to sphingosine-1-phosphate (S1P) [23,24]. Various cancer tissues, including prostate cancers, have a high expression of SPHK-1, which is associated with shorter survival [25,26,27,28,29,30,31].

Chrysin, also called 5, 7-dihydroxyflavone, is a flavone found in various natural products [32]. It has various pharmacological properties, including anticancer, neuroprotective, antiviral, antibacterial, anti-asthmatic, anti-inflammatory, hepatoprotective, nephroprotective, cardioprotective, antidiabetic, antidepressant, anxiolytic, and anti-arthritic activities [33,34]; however, the SPHK-1/HIF-1-mediated anticancer mechanism of chrysin has not been well studied. This study revealed a novel anticancer effect of chrysin in hypoxic PC-3 cancer cells as well as a PC-3 xenograft animal model.

## 2. Materials and Methods

### 2.1. Test Chemical

Chrysin (M.W. = 254.241 g/mol, purity ≥ 97% as determined through HPLC) was purchased from Sigma-Aldrich (Cat: C80105, St. Louis, MO, USA).

### 2.2. Cell Culture and Hypoxia Treatment

The PC-3 cells (human castration-resistant cancer cell line No. 21435, Korean cell line bank, Seoul, Korea) were cultured in RPMI 1640 (Cat: LM 011-01, Welgene, Daegu, Korea) medium supplemented with 10% fetal bovine serum (FBS) (Cat: S101-07, Welgene, Daegu, Korea) and 1% antibiotics (Cat: LS203-01, Welgene, Daegu, Korea). Normoxically conditioned cells were cultured in a 5% CO_2_ incubator at 37 °C. To create hypoxic conditions, the cells were cultured in a hypoxic chamber (Cat: NC9972080, Forma Scientific, Marietta, GA, USA) containing 1% O_2_, 5% CO_2_, and 94% N_2_ at 37 °C [35]; simulated hypoxic conditions were created by treatment with 150 µM cobalt chloride (CoCl_2_) (Cat: 232696, Sigma-Aldrich, St. Louis, MO, USA) [36,37].

### 2.3. 3D Culture

PC-3 cells (1 × 10^4^ cells/well) were seeded in 96-well plates (Cat: 90096, SPL, Pocheon, Korea) coated with 10 mg/mL of Matrigel (Cat: 356231, Corning, New York, NY, USA) and treated with chrysin (10 μM) for 48 h. Following spheroid formation, the cells were photographed at 20× magnification using a microscope (Nikon, Tokyo, Japan).

### 2.4. 3D Matrigel Embedded Culture (Long-Term Growth)

Cells were seeded in eight-well chamber glass plates (Cat: 30508, SPL, Pocheon, Korea) containing growth factor-reduced Matrigel. PC-3 cells (1 × 10^4^ cells/well) were maintained for 4 d in RPMI 1640 supplemented with 5% charcoal-stripped serum (Cat: A3382101, Thermo, Waltham, MA, USA) and 2% Matrigel; spheroid formation was observed within cells. Following their formation, the spheroids were treated with chrysin for 48 h. The spheroids were then photographed at 10× magnification using a microscope (Nikon, Tokyo, Japan).

### 2.5. Cell Viability Assay

Cell viability was evaluated using a CELLOMAX^TM^ Viability Kit (Cat: CM-VA2500, Precaregene, Hanam, Korea), and a microplate reader (Sunrise RC, Tecan, Mannedorf, Switzerland) was used to measure the absorbance. Various concentrations of chrysin under normoxic and hypoxic conditions were treated for 24 h in 96-well plates wherein cells were seeded (1 × 10^4^ cells/well). Cell viability was calculated using the following equation: cell viability (%) = (OD (chrysin) − OD (blank))/(OD (control) − OD (blank)) × 100.

### 2.6. Western Blot Analysis

Protein samples were prepared by cell lysis with radioimmunoprecipitation assay (RIPA) buffer (50 mM Tris-HCl at pH 7.4, 150 mM NaCl, 1% NP-40, 0.25% sodium deoxycholate, 1 M EDTA, 1 mM Na3VO4, 1 mM NaF, and protease inhibitor cocktail (Cat: 89900, 87786, 78420, Thermo, Waltham, MA, USA)) and were quantified using the Bio-Rad DC Protein Assay Kit II (Cat: 500-0113, 500-0114, 500-0115, Bio-Rad, Hercules, CA, USA). Quantified protein samples were loaded onto 8–15% sodium dodecyl sulfate polyacrylamide gel electrophoresis (SDS-PAGE) gels and were transferred at 250 mA for 2 h onto a Hybond ECL transfer membrane (Cat: 10600002, 10600001, Amersham Pharmacia, Piscataway, NJ, USA). Non-specific binding on membranes was blocked in fresh 5% non-fat dry milk (Cat: DI-232100, BD, Franklin Lakes, NJ, USA), after which the membranes were incubated with primary antibodies against SPHK-1 (ratio 1:1000, Cat: 3297, Cell Signaling, Danvers, MA, USA), HIF-1α (ratio 1:500, Cat: NB100-105, NOVUS, Littleton, CO, USA), AKT (ratio 1:1000, Cat: SC-8312, Santa Cruz, Dallas, TX, USA), p-AKT (ratio 1:1000, Cat: SC-7985, Santa Cruz, Dallas, TX, USA), GSK-3β (ratio 1:1000, Cat: 9832, Cell Signaling, Danvers, MA, USA), p-GSK-3β (ratio 1:1000, Cat: 9331, Cell Signaling, Danvers, MA, USA), Cyclin D1 (ratio 1:500, Cat: SC-8396, Santa Cruz, Dallas, TX, USA), VEGF (ratio 1:1000, Cat: SC-7269, Santa Cruz, Dallas, TX, USA), PARP (ratio 1:1000, Cat: SC-8007, Santa Cruz, Dallas, TX, USA), Bcl-2 (ratio 1:1000, Cat: SC-492, Santa Cruz, Dallas, TX, USA), cleaved caspase-3 (ratio 1:1000, Cat: 9664, Cell Signaling, Danvers, MA, USA), PCNA (ratio 1:3000, Cat: M0879, DAKO, Santa Clara, CA, USA), VE-cadherin (ratio 1:1000, Cat: ap2724a, Abcepta, San Diego, CA, USA), and β-actin (ratio 1:10000, Cat: A5316, Sigma-Aldrich, St. Louis, MO, USA) overnight at 4 °C. For the incubation secondary antibody on membranes, horseradish peroxidase (HRP)-conjugated anti-mouse (ratio 1:10000, Cat: 115-035-003, Jackson, West Grove, PA, USA) or anti-rabbit secondary antibodies (ratio 1:10000, Cat: 111-035-003, Jackson, West Grove, PA, USA) were used. An enhanced chemiluminescence (ECL) system (Cat: RPN2209, Amersham Pharmacia, Piscataway, NJ, USA) was used for protein expression. Densitometry data were obtained by quantifying each protein band using Image J 1.53k software (National Institutes of Health, Bethesda, MD, USA). The quantification of the protein levels was calculated from a duplicate analysis of each sample. The obtained protein levels of interest were normalized to β-actin.

### 2.7. Cell Cycle Assay

Cells treated for 24 h with 50 µM chrysin were washed and fixed with 70% ethanol overnight at −20 °C. The following day, the cells were treated with 10 mg/mL RNase A (Cat: RNASEA-RO, Roche, Basel, Switzerland) for 1 h at 37 °C. The cells were then stained with 1 mL of propidium iodide (PI) (50 µg/mL, Cat: P4170, Sigma-Aldrich, St. Louis, MO, USA). After filtering the cells using a 40 µm nylon mesh (Cat: 93040, SPL, Pocheon, Korea), the DNA content of the stained cells was analyzed using CellQuest software (BD) on a FACS caliber flow cytometer (BD).

### 2.8. Detection of Apoptosis Using CellEvent^TM^

Cells (1 × 10^4^ per well) were seeded in 96-well plates (SPL) and treated with 50 µM chrysin under normoxic and hypoxic conditions. After 24 h, 2 µM CellEvent^TM^ Caspase3/7 GREEN (Cat: C10423, Invitrogen, Carlsbad, CA, USA) was added to each well; all wells were then incubated for 40 min. Images were captured using a fluorescence microscope (Nikon Tokyo, Japan).

### 2.9. Vasculogenic Mimicry (VM) Tube-Formation Assay

A 24-well plate (Cat: 30024, SPL, Pocheon, Korea) was used to perform the VM tube-formation assay. Before seeding the cells, the well plates were polymerized with equal amounts of Matrigel and PBS at 37 °C for 1 h. The cells (3 × 10^5^) were added to coated wells in 10% FBS media or serum-free medium, following which they were incubated at 37 °C under hypoxic or normoxic conditions for 11 h. Formed tubes (i.e., tubular shapes) were counted after imaging using a light microscope Ts2_PH (Nikon, Tokyo, Japan) at 40× magnification.

### 2.10. Ethics Statement

The animal experiment was carried out according to the guidelines approved by the Institutional Animal Care and Use Committee of Kyung Hee University (KHUASP-16-072-1).

### 2.11. PC-3 Xenograft Model

Male BALB/c nude mice were purchased at five weeks old from DBL (Eumseong, Ko-rea). PC-3 cells (1 × 10^6^ cells/100 µL) were mixed with Matrigel (BD, 50% in 100 µL) and injected subcutaneously into the mice’s flanks. After 3 d, the mice were divided into three groups: (i) negative control group (*n* = 3), (ii) untreated control group (*n* = 6), and (iii) 50 mg chrysin/kg of body weight (*n* = 6). Chrysin or vehicle was orally administered to each mouse daily for three weeks. At the end of the experiment (i.e., 25 d after the start date of the animal study), all of the mice were sacrificed, and their tumors and blood were collected. The serum was subsequently separated from the collected blood through centrifugation.

### 2.12. Immunohistochemistry

Paraffin-embedded tumor tissue was cut into blocks of 5 µm thickness, after which the sections were then dewaxed and rehydrated; a 10 mM citrate buffer at pH 6.0 was used for antigen retrieval through microwaving. The sections were incubated for 30 min with a solution of 3% hydrogen peroxidase in methanol to block endogenous peroxidase, following which they were incubated in 6% horse serum in PBS for 1 h at room temperature to block non-specific binding. After this blocking, all sections were incubated with the primary antibodies (Ki-67 (ratio 1:200, Cat: ab16667, Abcam, Cambridge, UK), HIF-1α (ratio 1:50, Cat: NB100-105, NOVUS, Littleton, CO, USA), and VEGF (ratio 1:250, Santa Cruz, Dallas, TX, USA)) diluted in 1% BSA in PBS. Following washing, the sections were incubated with their corresponding secondary antibodies for 1 h at room temperature. The Vectastain ABC kit (Cat: PK-4002, Vector Laboratories, Burlingame, CA, USA) was used for the avidin–biotin complex (ABC) method, and peroxidase activity was visualized using 3,3-diaminobenzidine (Cat: K3468, DAKO, Santa Clara, CA, USA). All of the obtained slides were counterstained with hematoxylin and were then dehydrated and mounted; images were obtained using a light microscope. After avoiding necrotic areas, seven representative 200× power photomicrographs were taken with microscopic camera. The positively stained cells were counted for each image. The counting of the total stained cells was conducted with the ImagePro+ image-processing program.

### 2.13. Measurement of VEGF Production

The assessment of the VEGF levels in the serum was performed by using a VEGF ELISA Kit (Cat: KHG0112, Invitrogen, Carlsbad, CA, USA). VEGF production was measured according to the protocol provided by the company; the details have been described in our previous study [38].

### 2.14. siRNA Transfection

Control (Bioneer, Daejeon, Korea), SPHK-1 (Gene ID: 8877, Bioneer, Daejeon, Korea), and HIF-1α (Cat: SC-35561, Santa Cruz, Dallas, TX, USA) siRNAs were purchased from Bioneer and Santa Cruz. PC-3 cells were plated at a density of 5 × 10^5^ cells/well in a 60 mm cell culture dish. The cells were transfected with siRNA (25 pmol/well) using an siRNA transfection reagent (Cat: 409-10, Polyplus-transfection, Vectura, France) for 48 h. The cells were then treated with various concentrations of chrysin (i.e., 10 and 50 µM) under normoxic and hypoxic conditions, respectively. Finally, the cells were subjected to Western blotting.

### 2.15. Statistical Analysis

The data were expressed as the mean ± standard deviation (SD) of three replicates for each experiment in the study. One-way and two-way analysis of variance (ANOVA) was used to assess the significance of the differences between groups. Statistical significance was set at *p* < 0.05.

## 3. Results

### 3.1. Chrysin Reduced Tumor Spheroid Formation and Growth

**Figure 1:**
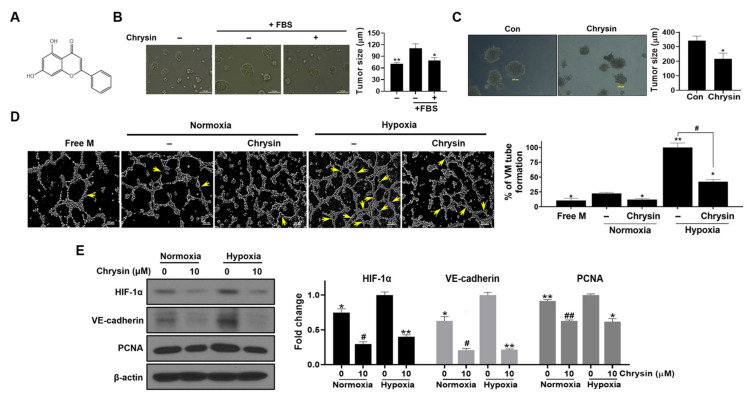
Effect of chrysin on 3D cell growth and vascular mimicry tube formation. (**A**) Chrysin chemical structure. (**B**) PC-3 cells (1 × 10^4^ cells/well) were seeded on a 96-well plate coated with Matrigel and treated with chrysin for 48 h. Spheroid formation was photographed at a 20× field using a microscope (Nikon, Tokyo, Japan). The bar graph indicates tumor size (in μm) (*n* = 3); * *p* < 0.05 and ** *p* < 0.01 when compared to the complete medium control group. (**C**) 3D Matrigel embedding culture (i.e., long-term growth). Cells were seeded into an eight-well chamber glass containing the reduced growth factor Matrigel. Cells were maintained for 4 d until spheroid formation, after which PC-3 spheroids were treated with chrysin for 48 h; spheroids were photographed at 40× field by microscope (Nikon, Tokyo, Japan). The bar graph indicates tumor size (in μm) (*n* = 3) and * *p* < 0.05 when compared to the untreated control group. (**D**) VM tube formation: PC-3 (3 × 10^5^) cells were seeded on coated wells in 10% FBS media or serum-free medium (Free M) and incubated at 37 °C under hypoxic or normoxic conditions for 11 h. The formed tubular network indicates a yellow arrow. Bar graph indicates percentage of VM tube formation (%). * *p* < 0.05 and ** *p* < 0.01 compared to normoxia control group. # *p* < 0.05 compared to hypoxia control. (**E**) Representative Western blot images and relative densitometric bar graphs of HIF-1α, VE-cadherin, and PCNA. The PC-3 cells were treated with chrysin or without for 24 h under normoxic or hypoxic conditions. Cell lysates were used for Western blotting to determine the expression of HIF-1α, VE-cadherin, PCNA, and β-actin. Data presented as the control’s fold change; * *p* < 0.05 and ** *p* < 0.01 when compared to the hypoxia control, while # *p* < 0.05 and ## *p* < 0.01 are values when compared to the normoxia control.

Chrysin has a ubiquitous 15-carbon flavone backbone with a molecular weight of 254.2 (Figure 1A). This study used a PC-3 tumor spheroid model to assess the effect of chrysin on tumor growth. The 3D culture model mimics some features of the in vivo tumor organization and is well suited for understanding the response of cancer cells to a drug. To investigate whether chrysin affects tumor formation, we treated the cells in Matrigel-coated wells with chrysin for 48 h. As shown in Figure 1B, PC-3 cells formed tumor spheroids up to 100 µm in diameter that were partly inhibited by chrysin. A second method involving 3D-spheroid growth was used to assess the inhibitory effect of chrysin on tumor growth following tumor spheroid formation. Chrysin was added for 48 h after 4 d of tumor spheroid formation. On day four after seeding, the PC-3 tumor spheroids reached 280–350 µm in diameter; the spheroids formed by chrysin-treated cells were smaller than those in the control PC-3 cells. The final average tumor spheroid diameters in the control and chrysin groups were 342 ± 30.7 and 217.3 ± 39 µm, respectively, on the sixth day after seeding (Figure 1C).

### 3.2. Chrysin Suppressed VM in PC-3 Cells under Normoxic and Hypoxic Conditions 

Chrysin inhibited tumor spheroid formation and growth (Figure 1). To examine whether chrysin affects VM, which is responsible for tumor growth in the tumor microenvironment, a PC-3 VM tube-formation assay was performed under normoxia and hypoxia. As shown in Figure 1D, the PC-3 cells formed tubular and sinusoidal networks under normoxia and hypoxia, although PC-3 tube formation was more pipe-like under hypoxia than that under normoxia. Chrysin treatment markedly inhibited PC-3 tube formation by 15% and 36% under normoxic and hypoxic conditions, respectively (Figure 1D). To examine the role of chrysin on the expression of VE-cadherin, which is associated with VM formation, and HIF-1α, this research analyzed the expression of these factors through Western blotting analyses. HIF-1α and VE-cadherin expression was found to be increased in PC-3 cells under hypoxic conditions compared to their expression in normoxic conditions. Chrysin downregulated the expression of HIF-1α and VE-cadherin in both normoxic and hypoxic conditions (Figure 1E).

### 3.3. Chrysin Inhibited Hypoxia-Induced Accumulation of SPHK-1 and HIF-1α in PC-3 Cells

**Figure 2:**
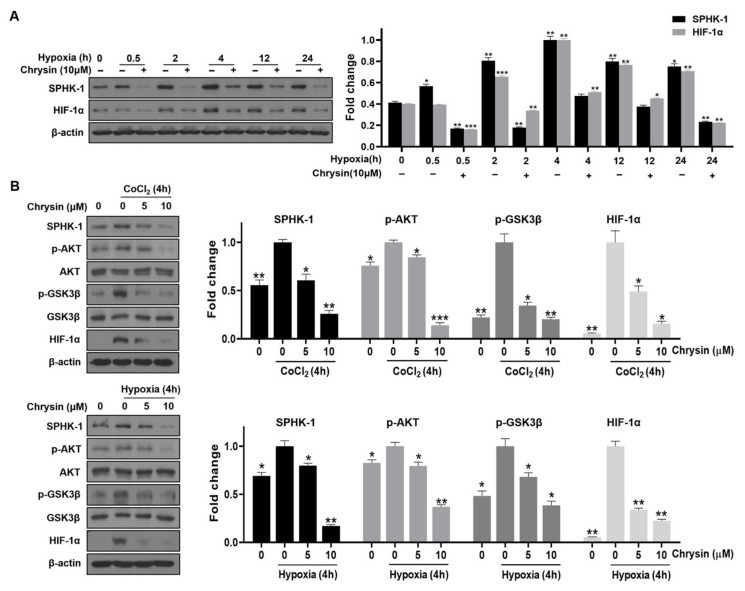
Effects of chrysin on the expression of SPHK-1 and its signaling proteins in hypoxic PC-3 cells. (**A**) Cells were treated with 10 μM chrysin for 0, 0.5, 4, 12, and 24 h under hypoxia. Western blotting was performed to determine SPHK-1 and HIF-1α expression. Quantitative protein levels are shown in the bar graph as mean ± SD for the duplicate. * *p* < 0.05, ** *p* < 0.01, and *** *p* < 0.001 compared with the hypoxia control. (**B**) Representative Western blot images and relative densitometric bar graphs of SPHK-1, AKT, p-AKT, p-GSK3β, GSK3β, and HIF-1α. Cells were treated with chrysin for 4 h under hypoxia or with the hypoxic mimic CoCl_2_. Western blotting was performed to determine SPHK-1, AKT, p-AKT, p-GSK3β, GSK3β, and HIF-1α expression. Bar graphs presented as a fold change of control. * *p* < 0.05, ** *p* < 0.01, and *** *p* < 0.001 when compared to the hypoxia control.

SPHK-1 is overexpressed in PC-3 cells and regulates HIF-1α upstream of the HIF-1α pathway. To investigate whether SPHK-1 is involved in chrysin-mediated HIF-1α inhibition, SPHK-1 and HIF-1α levels were analyzed by Western blotting in time-dependent hypoxic PC-3 cells. Western blot data showed that the accumulation of SPHK-1 and HIF-1α peaked 4 h following hypoxia exposure and then declined. In contrast, the expression of SPHK-1 and HIF-1α was consistently attenuated in chrysin-treated cells under hypoxic conditions (Figure 2A). As shown in Figure 2B,C, hypoxia and CoCl_2_ (i.e., a hypoxia mimic reagent) induced the phosphorylation of AKT and GSK-3β in PC-3 cells. Chrysin was found to significantly suppress SPHK-1, HIF-1α, and the phosphorylation of both AKT and GSK-3β in PC-3 cells both in hypoxia or after CoCl_2_ treatment (Figure 2B).

### 3.4. SPHK-1 Regulated HIF-1 Activation, and SPHK-1 Knockdown Contributed to Chrysin-Induced Anticancer Effects in Hypoxia

**Figure 3:**
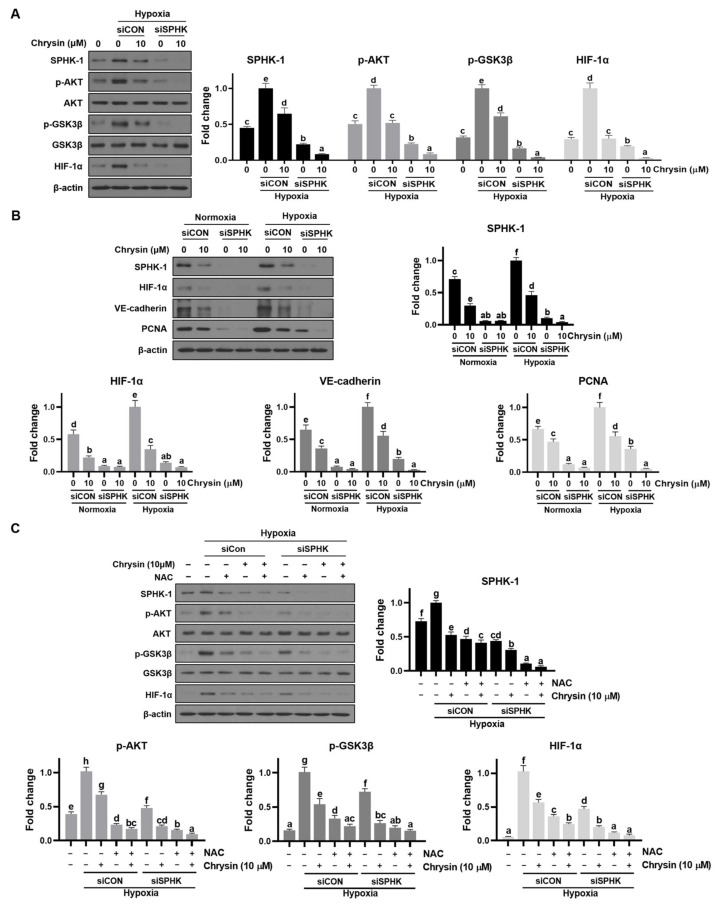
Effect of SPHK-1siRNA in chrysin-treated PC-3 cells. PC-3 cells were transfected with control or SPHK-1 siRNA for 48 h and were incubated in the presence or absence chrysin for 24 h. Cell lysates were prepared and subjected to Western blotting to analyze the expression. (**A**) Representative Western blot images and relative densitometric bar graphs of SPHK-1, p-AKT, p-GSK-3β, and HIF-1α. VE-cadherin, and PCNA. Bar graphs represent the quantification of interest protein related to β-actin or total AKT and total GSK3β, presented as a fold change of control. a~e means in a row by different superscripts are significantly different by LSD (least significant difference) at *p* < 0.05. (**B**) Representative Western blot images and relative densitometric bar graphs of SPHK-1, HIF-1α, VE-cadherin, and PCNA. Data represent mean ± SD for three times. a~f means in a row by different superscripts are significantly different by LSD (least significant difference) at *p* < 0.05. (**C**) Hypoxic PC-3 cells were treated with chrysin and/or NAC. Western blotting was performed to determine the expression of SPHK-1, HIF-1α, *p*-AKT, AKT, p-GSK-3β, GSK-3β, and β-actin in hypoxic PC-3 cells. Bar graphs represent the quantification of interest protein related to β-actin or total AKT and total GSK3β, presented as a fold change of control. a~h means in a row by different superscripts are significantly different by LSD (least significant difference) at *p* < 0.05.

To further confirm the role of SPHK-1 in the chrysin-mediated inhibition of AKT/GSK-3β signaling in hypoxia, SPHK-1 knockdown experiments were performed on hypoxic PC-3 cells. SPHK-1 knockdown was confirmed after SPHK-1 siRNA transfection using Western blotting, which promoted the inhibitory effects of chrysin on HIF-1α accumulation and phospho-AKT, and phospho-GSK3β in hypoxic PC-3 cells (Figure 3A). Additionally, SPHK-1 siRNA enhanced the inhibitory effect of chrysin on hypoxia-induced VE-cadherin and PCNA expression (Figure 3B). As ROS are associated with the SPHK-1/HIF-1α pathway [23,39] and chrysin is also known to have antioxidant activity, PC-3 cells were treated with chrysin and/or N-acetylcysteine (NAC) to examine whether ROS generation is associated with the chrysin-induced inhibition of HIF-1α and SPHK-1 expression. Chrysin or NAC treatment reduced hypoxia-mediated HIF-1α and SPHK-1 expression (Figure 3C). These results suggest that chrysin suppresses hypoxia-induced HIF-1α and SPHK-1 expression by inhibiting ROS-related pathways.

### 3.5. Chrysin Induced Apoptosis via Inhibition of SPHK-1/HIF-1α under Hypoxia

**Figure 4:**
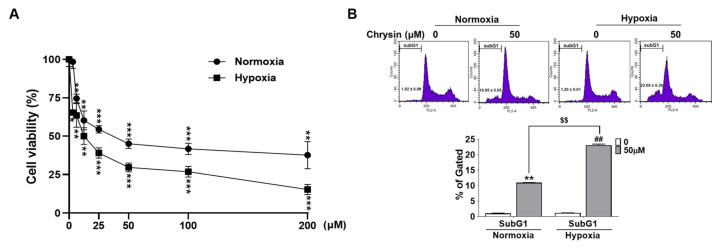

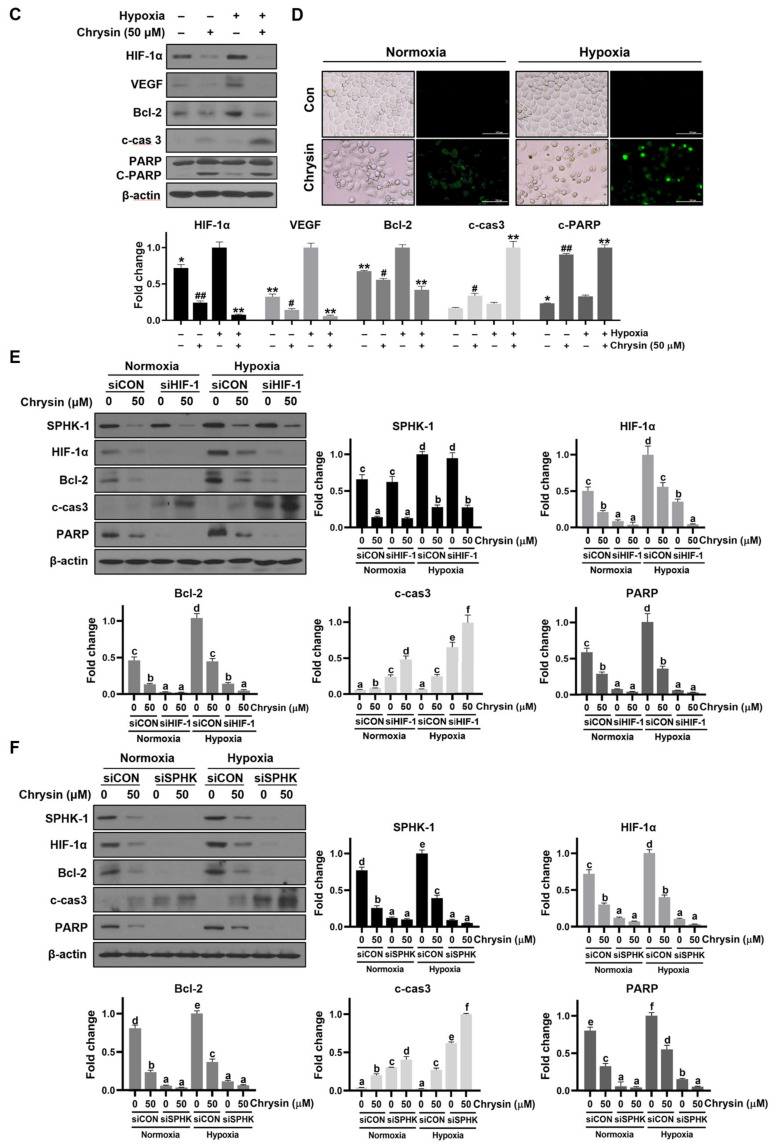
Effects of chrysin on apoptosis and the correlation between SPHK-1 and chrysin-induced apoptosis under hypoxia. (**A**) Effect of chrysin on the cytotoxicity of PC-3 cells for 24 h under normoxic and hypoxic conditions. Results are presented as the means ± SD of three independent experiments. * *p* < 0.05, ** *p* < 0.01, and *** *p* < 0.001 versus control groups. (**B**) Cells were used for FACS analysis to analyze their cell cycle. FACS image of cell cycle distribution diagram and bar graph. The bar graph expresses the percentage of sub-G1 population. Data represent mean ± SD values, ** *p* < 0.01 compared to normoxia control and ## *p* < 0.01 compared to hypoxia control and $$ *p* < 0.01 compared to normoxia chrysin-treated group. (**C**) Representative Western blot images and relative densitometric bar graphs of HIF-1α, VEGF, Bcl-2, c-cas-3, and c-PARP. Cells were treated with chrysin (50 μM) for 24 h under normoxia and hypoxia. The lysates were subjected to Western blotting for HIF-1α, VEGF, Bcl-2, c-cas-3, c-PARP, and β-actin. Bar graphs represent the quantification of interest protein related to β-actin and presented as a fold change of control; * *p* < 0.05 and ** *p* < 0.01 when compared to hypoxia control, while # *p* < 0.05 and ## *p* < 0.01 (compared to normoxia control). After the treatment of PC-3 cells with chrysin, (**D**) stained with CellEvent^TM^ (caspase-3/7) live dye. (**E**) Representative Western blot images and relative densitometric bar graphs of SPHK-1, HIF-1α, Bcl-2, c-cas-3, and PARP. PC-3 PCa cells were transfected with control or HIF-1α siRNA for 48 h and were incubated in the presence or absence of 50 μM chrysin for 24 h. Cell lysates were prepared and subjected to Western blotting to analyze the expression of SPHK-1, HIF-1α, Bcl-2, c-cas-3, PARP, and β-actin. Bar graphs represent the quantification of interest protein related to β-actin, presented as a fold change of control. a~f means in a row by different superscripts are significantly different by LSD (least significant difference) at *p* < 0.05. (**F**) Western blotting analysis on SPHK-1 siRNA-transfected and chrysin-treated samples. Bar graphs represent the quantification of interest protein related to β-actin and are presented as a fold change of control. a~f means in a row by different superscripts are significantly different by LSD (least significant difference) at *p* < 0.05.

A cell viability assay was performed to evaluate the cytotoxic effect of chrysin on PC-3 cells under hypoxia. The PC-3 cells were treated with various concentrations (i.e., 0, 3.15, 6.25, 12.5, 25, 50, 100, and 200 µg/mL) of chrysin under hypoxia for 24 h. Chrysin exerted significant cytotoxicity in normoxic and hypoxic PC-3 cells. Interestingly, chrysin-induced cytotoxicity was stronger under hypoxic conditions than under normoxic conditions. As shown in Figure 4A, the viability of the PC-3 cells was reduced to 55% and 70% when incubated with 50 µM chrysin for 24 h in normoxia and hypoxia, respectively, for 24 h. To confirm that chrysin induces apoptosis, the activation of caspase-3 and inhibition of Bcl-2, key molecules in the apoptotic pathway, was evaluated in PC-3 cells under both normoxic and hypoxic conditions using Western blotting. As expected, chrysin induced the cleavage of caspase-3 and PARP under both conditions. Exposure to hypoxia led to increased levels of VEGF, HIF-1α, and Bcl-2. The induced-VEGF and HIF-1α expression was reduced by chrysin treatment (Figure 4C). Consistent with the cell toxicity data (Figure 4A), chrysin-induced apoptosis was more active in hypoxia than in normoxia. In addition, cell cycle analysis showed an increased accumulation of sub-G1 cells following chrysin treatment from 1.02% to 10.95% and 23.08% under normoxia and hypoxic conditions, respectively (Figure 4B). As shown in Figure 4D, microscopic examinations revealed that chrysin-treated PC-3 cells exhibited increased apoptotic morphological features including cell shrinkage and apoptotic bodies. A significant increase in CellEvent^TM^ caspase-3/7-positive PC-3 cells was observed following chrysin treatment under normoxia and hypoxia. The detection of apoptosis using a fluorescent agent further confirmed that hypoxia enhanced chrysin-induced apoptotic cell death, which was consistent with the cell cycle data (Figure 4D). To investigate whether the inhibitory effect of chrysin on SPHK-1/HIF-1α is associated with apoptotic induction in PC-3 cells under hypoxia, we knocked down HIF-1α and SPHK-1 in PC-3 cells using the corresponding siRNAs and analyzed the apoptotic markers using Western blotting under hypoxic conditions (Figure 4E,F). As shown in Figure 4E and F, the knockdown of HIF-1α and SPHK-1 reduced Bcl-2 and total PARP expression, and increased the cleavage of caspase-3. The combined treatment with chrysin and SPHK-1 or HIF-1α siRNA had a greater effect on the apoptotic markers than the single treatments, indicating that HIF-1α/SPHK-1 inhibition is important for chrysin-induced apoptosis in PC-3 cells under hypoxic conditions.

### 3.6. Chrysin Inhibited PC-3 Xenograft Growth in Nude Mice 

**Figure 5:**
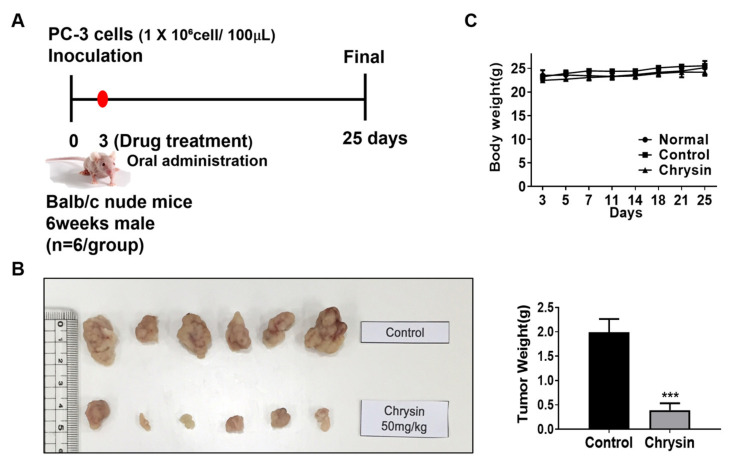
PC-3 xenograft growth-suppressing activity of chrysin in athymic nude mice. (**A**) Diagram of animal study plans and time points; PC-3 cells (1 × 10^6^ cells/100 µL) were mixed with Matrigel (BD, 50% in 100 µL) and were injected subcutaneously into mice’s flank. After 3 days, chrysin or vehicle was orally administered daily for three weeks. (**B**) Final tumor weight at termination of the experiment. Photographs of dissected tumors. Values are mean ± SD, *n* = 6, and *** *p* < 0.001 compared to control. (**C**) Body weights of mice.

To confirm the in vivo efficacy of chrysin and to obtain tissues for analyzing the biomarkers of efficacy and molecular targets, we evaluated the effect of chrysin on PC-3 xenograft growth in athymic BALB/c nude mice. Chrysin (50 mg/kg) was administered orally 3 d after the injection of cancer cells into mice flanks and continued as daily gavage treatment for 25 d, following which all of the mice were sacrificed (Figure 5A). The chrysin-treated group showed a significant inhibition of tumor growth and a reduction in tumor weight (by 80% at necropsy) (Figure 5B); this did not have any adverse effects on the body weight of the test mice (Figure 5C).

### 3.7. SPHK-1/HIF-1 Suppression Associated with Anticancer Effect in Chrysin-Treated Xenograft

**Figure 6:**
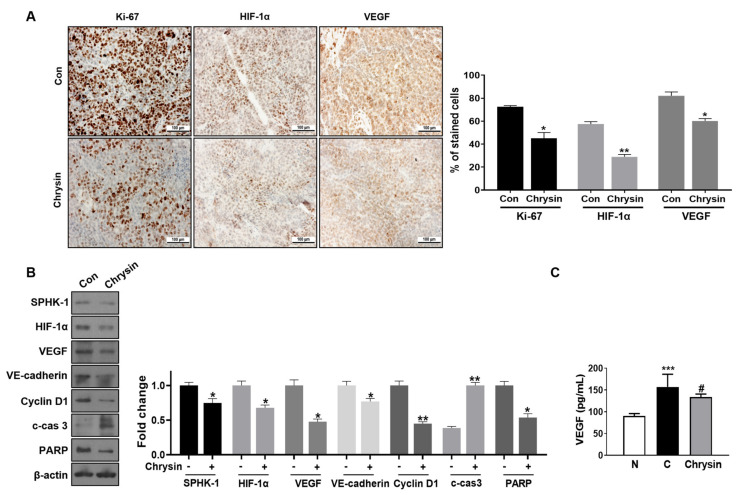
Anti-cancer effects of chrysin in the proposed mouse xenograft mouse model. (**A**) Representative specimen of immunohistochemical staining for Ki-67, HIF-1α, and VEGF and bar graph expressing the Ki-67, HIF-1α, and VEGF percentage of stained cells (%) in tumor sections. Data represent mean ± SD; *n* = 6/group. * *p* < 0.05, and ** *p* < 0.01 (compared to control group). (**B**) Western blotting for SPHK-1, HIF-1α, VEGF, VE-cadherin, Cyclin D1, c-cas3, PARP, and β-actin of selected tumors. Bar graphs represent the quantification of interest protein related to β-actin, presented as a fold change of control. * *p* < 0.05 and ** *p* < 0.01 when compared to untreated control. (**C**) The concentration of VEGF in the serum samples of nude mice determined by ELISA. Data represent mean ± SD; *n* = 6/group. *** *p* < 0.001 (compared to normal control (N)), and # *p* < 0.05 (compared to control (C)).

The IHC analysis showed that the chrysin-treated tumors contained significantly decreased amounts of the proliferation marker Ki-67 (27% inhibition compared to the control group) (Figure 6A). Fewer HIF-1α-positive cells were observed in the chrysin-treated tumors than in the control group tumors (Figure 6A). VEGF expression showed cytosolic expression, and the control group showed intense staining of 80% of the cytosol. In contrast, the chrysin-treated group showed a 20% reduction in VEGF expression compared with the control group (Figure 6A). Western blot analyses of the tumors obtained from the control and chrysin-treated mice showed the suppression of SPHK-1, HIF-1α, cyclinD1, VE-cadherin, and total PARP, as well as increased cleavage of caspase-3 in chrysin-treated tumors (Figure 6B). Serum VEGF levels showed that the control group had the highest levels among the three groups (normal, control, and chrysin-treated group). The serum VEGF levels in the chrysin-treated group were lower than those in the control group (Figure 6C).

## 4. Discussion

Hypoxia is a typical feature of most solid tumors. Recent research has shown that the hypoxic microenvironment regulates the various pathways involved in tumor progression, angiogenesis, and VM [40]. HIF-1α, the main transcription factor involved in hypoxia, is overexpressed in many types of cancer. HIF-1α overexpression activates genes associated with tumor development and aggression and is associated with a poor prognosis [41]. The association of HIF-1α expression with clinicopathological significance has recently been reported in various cancer types, including prostate cancer [42,43,44,45]. The study of HIF-1α as a target for cancer chemotherapy has thus been reported in ≥7500 studies and has progressed rapidly. Pathways related to HIF-1α represent potential targets for cancer therapy.

The researchers of this paper have been studying natural products and active compounds from natural products that target SPHK-1/HIF-1α signaling. We previously found that pristimerin inhibits SPHK-1/HIF-1α signaling [38]. In this study, the SPHK-1/HIF-1α pathway was identified as a potential therapeutic target for aggressive prostate cancer treatment. SPHK-1 is an HIF-1α regulator that modulates HIF-1 stability during hypoxia through pVHL-dependent proteasomal degradation [23,46,47,48]. Consistently, this study confirmed that hypoxia significantly increased the accumulation of SHPK-1 and HIF-1α in cells (Figure 2A). Furthermore, hypoxia-induced HIF-1α accumulation was significantly suppressed in the presence of chrysin, consistent with the results of previous studies [49].

Interestingly, chrysin was found to significantly reduce the SPHK-1 expression and activity (Figure S1) in PC-3 cells under hypoxic conditions. In addition, chrysin has been tested in hypoxia-induced SPHK-1 and HIF-1α expression in hypoxic DU145 cells, another CRPC cell line. It was confirmed that chrysin decreases SPHK-1 and HIF-1α in the same way as those of PC-3 cells (Figure S2). To further confirm the involvement of SPHK-1 in the chrysin-induced inhibition of HIF-1α under hypoxia, we assessed the effects of chrysin on the phosphorylation of AKT and GSK-3β, as SPHK1-dependent stabilization of HIF-1α is known to be mediated by the AKT/GSK-3 signaling pathway [22,23]. Our data demonstrated that chrysin significantly inhibited the hypoxia-mediated phosphorylation of AKT and GSK3β in PC-3 cells under both hypoxia and CoCl_2_ treatment (Figure 2B). Additionally, SPHK-1 siRNA transfection enhanced the inhibitory effect of chrysin on HIF-1α accumulation and phospho-AKT and GSK3β levels in PC-3 cells (Figure 3A). We also showed that chrysin inhibits SPHK-1 and HIF-1α expression in tumor tissues (Figure 6A,B).

Recently, a large number of studies have reported that hypoxia and HIF molecules promote VM in various cancers [50,51,52]. HIF-1 directly modulates the molecules associated with VM, such as VEGF, VE-cadherin, LOX, Twist, MMP2, and MIFect [53,54]. A few studies have provided evidence that SPHK-1 mediates VM by regulating VEGF expression, one of the key genes regulated by HIF-1α [55], and that VE-cadherin is responsible for VM by controlling S1P and S1PR1 [56]. Furthermore, SPHK-1-positive expression is significantly associated with VM and poor survival in colorectal cancer [57]. Interestingly, our data demonstrated that hypoxia enhanced VM with increased expression of HIF-1α, SPHK-1, VE-cadherin, and VEGF (Figure 1D,E, Figure 3B, and Figure 4C). To confirm the involvement of SPHK-1 in the chrysin-induced inhibition of VE-cadherin in hypoxic conditions, we assessed the effects of chrysin on SPHK-1 siRNA-transfected cells. Transfection with SPHK-1 siRNA enhanced the inhibitory effect of chrysin on VE-cadherin in PC-3 cells (Figure 3B). Similarly, animal data collected during this study showed that chrysin treatment reduced VE-cadherin and VEGF expression in tumor tissues (Figure 6B). Consistently, VEGF expression decreased in the serum of chrysin-treated mice in the PC-3 xenograft model (Figure 6C).

In the present study, chrysin inhibited the growth of PC-3 cells in 2D and 3D cultures and tumor growth in a PC-3 xenograft model. We found that the growth inhibitory effect of chrysin was associated with the induction of apoptosis, as evidenced by the increase in the sub-G1 population, the cleavage of caspase-3 and PARP, and the decrease in Bcl-2. The oncogenic functions of SPHK-1 and HIF-1α have been linked to the promotion of cell proliferation, survival, and the induction of resistance to apoptosis. Thus, to confirm the involvement of SPHK-1 and HIF-1α in the chrysin-mediated induction of apoptosis, hypoxic PC-3 cells were treated with SPHK-1 and HIF-1α siRNAs during this study. Interestingly, co-treatment with SPHK-1 and HIF-1α siRNA was even found to induce apoptosis-related proteins, such as cleaved caspase-3, and inhibit anti-apoptotic proteins, such as total PARP and Bcl-2 (Figure 4E,F).

## 5. Conclusions

In summary, this study showed that chrysin inhibits tumor growth and VM by inhibiting HIF-1α, SPHK-1, and phospho-AKT/GSK-3β signaling in PC-3 cells under hypoxia. Moreover, chrysin induced apoptosis-related markers and decreased the expression of VE-cadherin and VEGF. Chrysin was thus confirmed to inhibit cancer growth in vivo in this research. These findings suggest that chrysin exerts anticancer effects by suppressing HIF-1α accumulation via SPHK-1.

## Supplementary Materials

The following supporting information can be downloaded at: https://www.mdpi.com/article/10.3390/cells11182787/s1. Supplementary Figure S1: Effect of chrysin on the SPHK-1 activity in normoxic or hypoxic PC-3 cells; Figure S2: Representative Western blot images and relative densitometric bar graphs of SPHK-1 and HIF-1α; Figure S3: Effect of chrysin on SPHK-1, HIF-1α, and VEGF in S1P-induced PC-3 cells.
