# Supplementary material for: SPHK/HIF-1α Signaling Pathway Has a Critical Role in Chrysin-Induced Anticancer Activity in Hypoxia-Induced PC-3 Cells
Source: Cells. 2022 Sep 7;11(18):2787. doi: 10.3390/cells11182787 (PMC9496844; doi:10.3390/cells11182787)
Supplement: Supplementary file 1 [file cells-11-02787-s001.zip › cells-1808584-supplementary.pdf]

cells MDPI

SUPPLEMENTARY FIGURES and LEGENDS

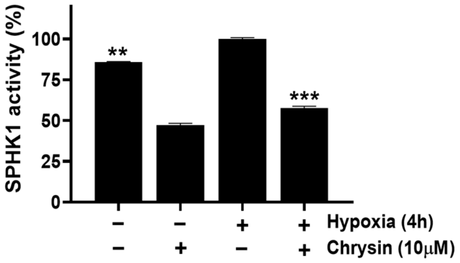

Figure S1. Effect of chrysin on the SPHK-1 activity in normoxic or hypoxic PC-3 cells. Cells were treated with 10 μM chrysin for 4 h under normoxia and hypoxia. Sphingosine kinase activity was measured by using sphingosine kinase activity assay kit (Cat: K-3500, Echelon, Salt Lake City, UT, USA) according to the manufacturer's instructions. In brief, protein extracts (30 μg) were incubated in reaction buffer (100 μM sphingosine and 10 μM ATP) for 1 h at 37 °C, and luminescence attached ATP detector was added to stop the kinase reaction. Kinase activity was measured using Lumistar Optima luminometer (BMG LABTECH, Offenburg, Germany). Quantitative SPHK-1 activity levels are shown in bar graphs as mean ± SD for the duplicate. $^{**}p < 0.01$ and $^{***}p < 0.001$ compared hypoxia control.

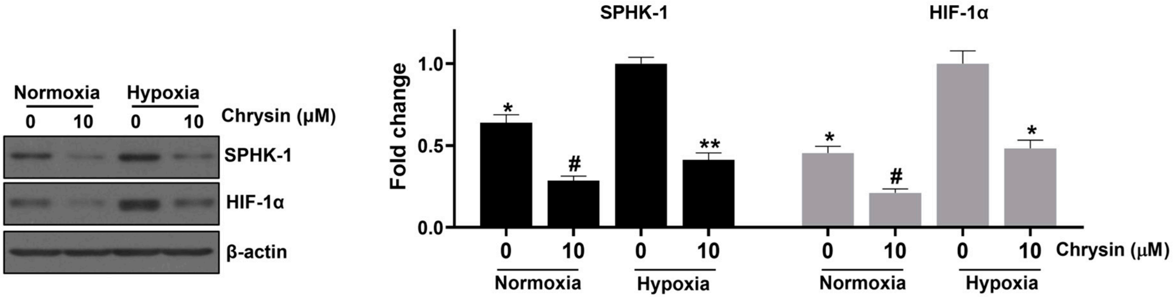

Figure S2. Representative western blot images and relative densitometric bar graphs of SPHK-1 and HIF-1α. Chrysin decreases SPHK-1 and HIF-1α in hypoxic or normoxic DU145 cells. The DU145 cells were cultured in RPMI 1640 medium supplemented with 10% fetal bovine serum and 1% antibiotics. DU145 cells (5 × 10^5 cells/well) were seeded in a 60 mm cell culture dish and treated with chrysin (10 μM) under normoxic or hypoxic conditions for 24 h. Cell lysates were prepared and subjected to western blotting to analyze the expression of SPHK-1, HIF-1α, and β-actin. Bar graphs represent the quantification of interest protein related to β-actin and present as a fold change of control. $^{*}p < 0.05$ and $^{**}p < 0.01$when compared to the hypoxia control. $\#p < 0.05$ is value when compared to normoxia control.

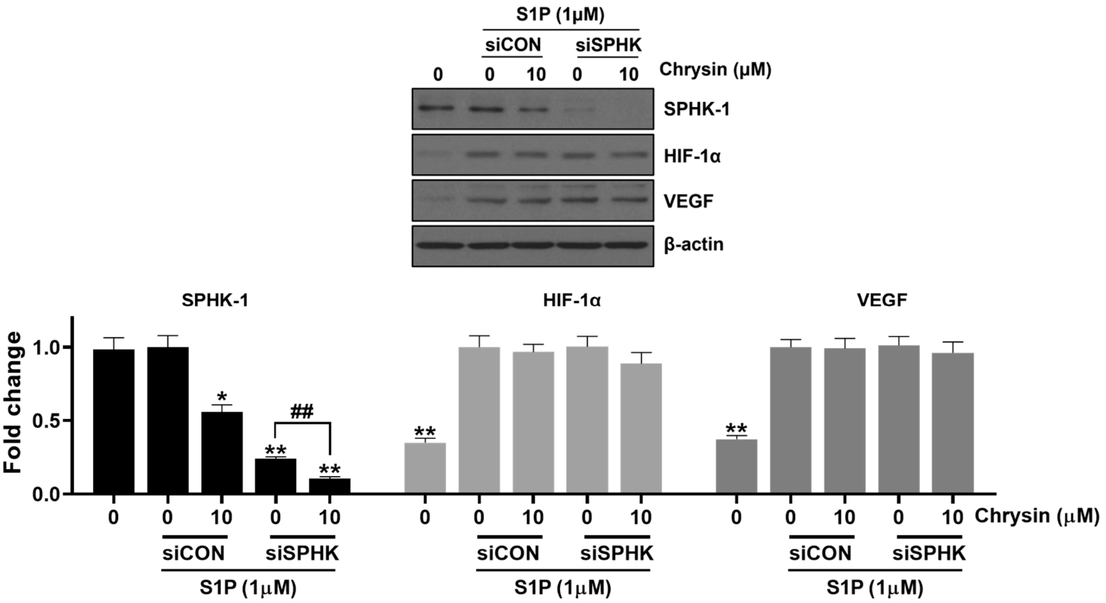

Figure S3. Effect of chrysin on SPHK-1, HIF-1α, and VEGF in S1P- induced PC-3 cells. Representative western blot images and relative densitometric bar graphs of SPHK-1, HIF-1α, and VEGF. The cells were treated with S1P(1μM) or chrysin (10μM) for 24h after transfection SPHK-1 siRNA (48 h). Cell lysates were prepared and subjected to western blotting to analyze the expression of SPHK-1, HIF-1α, VEGF , and β-actin. Bar graphs represent the quantification of interest protein related to β-actin, as fold change of control. *$p < 0.05$ and **$p < 0.01$ are values when compared with S1P control. ##$p < 0.01$ is value when compared with SPHK1 siRNA(siSPHK) control.
